# Cerebellum and Prematurity: A Complex Interplay Between Disruptive and Dysmaturational Events

**DOI:** 10.3389/fnsys.2021.655164

**Published:** 2021-06-10

**Authors:** Giulia Spoto, Greta Amore, Luigi Vetri, Giuseppe Quatrosi, Anna Cafeo, Eloisa Gitto, Antonio Gennaro Nicotera, Gabriella Di Rosa

**Affiliations:** ^1^Unit of Child Neurology and Psychiatry, Department of Human Pathology of the Adult and Developmental Age “Gaetano Barresi”, University of Messina, Messina, Italy; ^2^Department of Health Promotion, Mother and Child Care, Internal Medicine and Medical Specialties (ProMISE), University of Palermo, Palermo, Italy; ^3^Neonatal Intensive Care Unit, Department of Human Pathology of the Adult and Developmental Age “Gaetano Barresi”, University of Messina, Messina, Italy

**Keywords:** cerebellar hemorrhage, cerebellar infarction, cerebellar underdevelopment, cerebellum and neurodevelopment, early intervention, prematurity

## Abstract

The cerebellum plays a critical regulatory role in motor coordination, cognition, behavior, language, memory, and learning, hence overseeing a multiplicity of functions. Cerebellar development begins during early embryonic development, lasting until the first postnatal years. Particularly, the greatest increase of its volume occurs during the third trimester of pregnancy, which represents a critical period for cerebellar maturation. Preterm birth and all the related prenatal and perinatal contingencies may determine both dysmaturative and lesional events, potentially involving the developing cerebellum, and contributing to the constellation of the neuropsychiatric outcomes with several implications in setting-up clinical follow-up and early intervention.

## Introduction

In the last few decades, increasing evidence has driven attention to the cerebellar role in motor coordination as initially believed, and in cognition, behavior, language, memory, and learning (Salman and Tsai, 2016). Although adult cerebellar morphology and basic circuitry have been described for more than 100 years, the molecular and cellular mechanisms which drive cerebellar development have more recently begun to be elucidated (Haldipur et al., 2018). Human cerebellar development occurs late in gestation and is hindered by preterm birth. Moreover, longitudinal studies investigating cerebellar maturational trajectories from birth through childhood in preterm infants have revealed smaller volumes and reduced cerebellum growth both at short- and long-term observation. Beyond gestational age, several perinatal factors have been associated with smaller cerebellar volumes and growth (Pieterman et al., 2018). Encephalopathy of the preterm encompasses a constellation of neuropathological and clinical signs originating from both dysmaturative and lesional events occurring during prenatal and perinatal life, obviously including cerebellar development (Matthews et al., 2018). Indeed, it is reported that multiple perinatal risk factors, including exposure to invasive procedures in Neonatal Intensive Care Units (NICUs), may influence the central nervous system (CNS) maturation, leading to poor neurodevelopmental outcomes (Valeri et al., 2015; Montirosso et al., 2016). Here we reviewed the main morphological and neurodevelopmental features of cerebellar dysfunctions associated with preterm birth and their implications in setting-up clinical follow-up and early intervention.

## Cerebellar Development and Prematurity

The human cerebellum develops over a long time, extending from early embryonic period until the first postnatal years. To date, it is commonly accepted that the first cerebellar anlage starts to form in the first month of gestation, and lasts until the second postnatal year (van Essen et al., 2020). However, most of its volume increases during the last trimester of pregnancy, which is considered a critical period for cerebellar development (Volpe, 2009).

The cerebellar anlage takes origin from the hindbrain due to the interaction of multiple embryonic structures (ten Donkelaar et al., 2003). The first step of this process consists in the expression of the transcription factors Otx2 and Gbx2 that allow the organization of the forebrain/midbrain and hindbrain territories, respectively, determining a boundary structure called Isthmic organizer (Marin and Puelles, 1994; Liu et al., 1999). This is a patterning center essential for the establishment of the anterior-posterior axis and the rhombomere segmentation (De Luca et al., 2016; van Essen et al., 2020), and a disruption of its organization in the earliest period of cerebellar development may result in cerebellar hypoplasia (CH) with disproportionally hypoplastic vermis (Basson and Wingate, 2013).

Later, in rhombomere 1, the proteins Atoh1 and Ptf1a identify the rhombic lip and the ventricular zone, respectively, the areas including the cerebellar progenitors: the rhombic lip will originate the glutamatergic cells, while the ventricular zone will produce GABAergic cells (Hoshino et al., 2005; Machold and Fishell, 2005; Wang et al., 2005). The granule layer cells are glutamatergic neurons originating from the granule cell progenitors (GCPs), a population that migrates tangentially to form the external granule layer during the last months of pregnancy and undergoes a cloning expansion, eventually giving rise to 95% of all cerebellar neurons.

Being the most represented cell type in the cerebellum, a disorder in this fast and massive proliferation might determine a severe hypoplasia (Basson and Wingate, 2013). This overgrowth process is mediated by the expression of Sonic Hedgehog (SHH), a protein produced by mature Purkinje cells, Bergmann glia, and choroid plexus cells (De Luca et al., 2016; Cheng et al., 2018). Therefore, a defect in the SHH signaling pathway may cause a reduction in the entire cerebellar volume, inducing a more homogeneous hypoplasia (Basson and Wingate, 2013).

During the third trimester of pregnancy, the Purkinje cells (the GABAergic neurons deriving from the ventricular zone) are organized in a single layer and begin to make connections in the molecular layer with the parallel fibers and the stellate cells (Leto et al., 2016). This maturation occurs between the 24th and the 40th gestational week, determining overall a fivefold dimensional enlargement of the cerebellum and its subsequent foliation to accommodate in the posterior fossa (Volpe, 2009).

The last few months of gestation are also the time during which the cerebellar nuclei start to form their major afferents and efferences from/to the cerebral cortices and subcortical structures, i.e., thalamus, and this development will continue during the first years after birth, inducing the expansion of the cerebellar anlage (Pierson and Al Sufiani, 2016; Sathyanesan et al., 2019).

The principal afferent pathways of the cerebellum are the mossy fibers and the climbing fibers. The former originates from the brainstem nuclei, the spinal cord and the reticular formation, and the latter from the inferior olivary complex (Roostaei et al., 2014). Conversely, the deep nuclei represent the cerebellum’s outputs, through which the efferences deriving from the vermis and the hemispheres get to their targets (Kandel et al., 2013). The archicerebellum’s efferences (corresponding to the flocculonodular lobe) constitute an exception since they reach their target (vestibular nuclei), bypassing the deep nuclei (Roostaei et al., 2014). In detail, the vermis receives visual, auditory and vestibular inputs and, together with the intermediate zones of the hemispheres (paravermis), welcomes sensorimotor information.

The efferences coming from these two zones (globally known as spinocerebellum) mainly target the medial vestigial and interposed nuclei, giving origin to the descending motor system. The lateral zones of the hemispheres (cerebrocerebellum) are reciprocally connected to the cerebral cortex, and their outputs target the dentate nucleus (Basson and Wingate, 2013; Roostaei et al., 2014). On the whole, it is reasonable to state that “for the majority of the cerebellum, the targeting of output of each nucleus determines the functional output of the overlying cerebellar cortex” (Basson and Wingate, 2013), acknowledging a correspondence between the anatomical and functional organization.

Nowadays, numerous studies have clearly established the cerebellar connections with the contralateral cerebral cortices and particularly with the dorsolateral prefrontal cortex, the parietal and superior temporal lobes (Dijkshoorn et al., 2020). An interruption of this well-formed circuit between areas of the CNS may determine a diaschisis, meaning a functional impairment of the region linked to the one subject of a structural lesion (Catsman-Berrevoets, 2017). Therefore, it may be clearly assumed that damage of CNS territories correlated to the forming cerebellum may also alter its morphological architecture due to a trans-synaptic degeneration (Volpe, 2009).

The disruption of the cerebral and cerebellar white matter due to prematurity and its complications, in association with supratentorial injuries or even in their absence, has been investigated by relatively new neuroimaging techniques, such as tractography. In that regard, Hasegawa et al. (2018) showed a reduction of the fractional anisotropy in the superior cerebellar peduncle (through which the efferences are sent from the cerebellum to the cerebral cortex), and in the medial one (through which the cerebellum receives auditory, visual, vestibular, and somesthetic afferences); whereas, Brossard-Racine et al. (2017) revealed an increase of the fractional anisotropy in the dentate nuclei. Moreover, a lower mean diffusivity in the vermis has been reported and correlated to the severity of supratentorial injuries (Brossard-Racine et al., 2017).

## Cerebellar Neuropathology

Cerebellar injury in preterms has been a subject of interest for many years. Indeed, preterm cerebellar lesions can be divided into two main groups: (1) cerebellar underdevelopment (namely cerebellar atrophy/hypoplasia, which may be regional or global); and (2) destructive cerebellar lesions (which are primary and focal injury, manifesting as hemorrhage or infarction) (Volpe, 2009; Tam, 2018).

### Cerebellar Underdevelopment

To date, cerebellar underdevelopment (hence atrophy and hypoplasia) is one of the most common complications in preterm infants associated with poor neurodevelopmental outcome (Gano and Barkovich, 2019). In particular, hypoplasia refers to a structure exhibiting incomplete development or underdevelopment, often due to a developmental arrest, whereas atrophy is due to degeneration of previously existing cells in a formed structure that results in decreased organ or tissue size. Since both of these manifestations lead to a decreased cerebellar volume, it is difficult to discriminate between these two entities; hence in literature, they are usually referred to as a unique condition (Pierson and Al Sufiani, 2016; Poretti and Boltshauser, 2015).

Cerebellar hypoplasia after prematurity has been reported for the first time in a study carried out by Allin et al. (2001), who detected a reduction in cerebellar volume in a group of adolescents born before the 33rd week of gestation, compared to a control group. It is noteworthy mentioning that CH may globally involve the cerebellum (affecting equally the hemispheres and the vermis) or, in contrast, may present with a volume loss of both the hemispheres with or without vermis abnormalities (Tam, 2018). In this regard, a recent study carried out by Wu et al. (2020) compared preterm babies and healthy controls, demonstrating that prematures show not only smaller volumes (global and regional), but also different shapes both of cerebellum and brainstem, even when a structural injury of these areas fails to be detected by MRI scans. Even if numerous lesional patterns have been reported, the most typically observed includes bilateral and symmetric involvement of the two cerebellar hemispheres, associated with smaller pons, and supratentorial injuries (Pierson and Al Sufiani, 2016). However, it is also necessary to remember that 25% of CH coexisting cases with pontine hypoplasia are ascribable to genetic causes (Poretti et al., 2014). These conditions must be taken into account during the assessment and the differential diagnosis of CH. Nevertheless, they will not be discussed in this review, as they may determine *per se* CH, regardless of preterm birth’s potential coexistence.

Currently, many factors are known to be potential causes of cerebellar underdevelopment in preterm babies. Among these, the most significant include blood products (hemosiderin), perinatal glucocorticoid exposure, opioids and pain, inadequate nutrition, infections, inflammations, hypoxic-ischemic insults, cerebral brain injuries, and socioeconomic status (Volpe, 2009; Tam, 2018).

#### Blood Products (Hemosiderin)

Blood products derive from different types of hemorrhages, either directly involving the cerebellum (intraparenchymal hemorrhages) or adjacent structures (intraventricular and subarachnoid hemorrhages). Whilst it is true that cerebellar hemorrhages (CBHs) may result in a destructive lesion (which will be later discussed), blood products *per se*, particularly hemosiderin, may determine CH. This may be due to hemosiderin’s direct effect, which reaches the surface of the cerebellum and the brainstem, traveling inside the cerebrospinal fluid and generating reactive oxygen species (ROS) (Volpe, 2009; Gano and Barkovich, 2019). The GCPs of the external granular layer appear to be highly susceptible to ROS-mediated damage and, once struck, may lead to cerebellar underdevelopment (Volpe, 2009; Gano and Barkovich, 2019). As a consequence, obstructive hydrocephalus may occur, which, in its turn, could lead to further mechanical injuries to the CNS (Tam, 2018). Another proposed mechanism through which hemosiderin may lead to CH implies a dysfunction of the FOXC1 pathway, usually responsible for the embryonic cerebellar growth *via* mesenchymal-dependent signaling (Haldipur et al., 2014). Moreover, another potential mechanism, though to the best of our knowledge not yet demonstrated, may involve the SHH pathway, known for its proliferative effect on the external GCPs. Usually, an alteration of this specific pathway is described in literature regarding glucocorticoid exposure (Heine and Rowitch, 2009). Therefore, further investigation on this potential correlation might be of interest for future research.

#### Glucocorticoid Exposure

Perinatal exposure to glucocorticoids may have adverse effects on the developing CNS, potentially leading to poor neurodevelopmental outcomes. However, there are a wide variety of conditions requiring glucocorticoid administration, both in the prenatal and postnatal periods. Antenatally, betamethasone or dexamethasone are the most used glucocorticoids in the mother at high risk of preterm delivery to promote lung maturation. Postnatally, dexamethasone is used to avoid or treat chronic bronchopulmonary diseases, while hydrocortisone is commonly employed in managing refractory hypotension in premature newborns (Tam, 2018). A recent case-series emphasized the use of hydrocortisone to treat refractory neonatal seizures (Di Rosa et al., 2020). In particular, a clear model of long-term effect on the brain volume played by glucocorticoids can be provided by the adrenocorticotropic hormone, the gold standard treatment for infantile spasms. In this case, the brain volume loss appeared to be proportional to brain’s immaturity (Salpietro et al., 2014). According to these assumptions, the fetal age, during which the cerebellum is notably still growing, can be considered the most “at-risk.” Moreover, the highest concentrations of glucocorticoid receptors in the brain have been reported in prenatal and early postnatal life (Pavlík and Buresová, 1984). Experimental studies showed that glucocorticoids might interfere with normal neurodevelopment through mechanisms that involve transcription factors and protease enzymes (Aden et al., 2008; Noguchi et al., 2008; Bhatt et al., 2013; Austdal et al., 2016). This way, the proliferation of the progenitor brain cells (namely the GCPs in the cerebellum) may be impaired, resulting in apoptosis and loss of neural function. The glucocorticoids-mediated inhibition of Sonic-Hedgehog-Smoothened signaling, notably involved in the proliferation of the GCPs (Heine and Rowitch, 2009) has been thought to underlie cerebellar dysfunction. Conversely, the interplay between SHH and glucocorticoids has been further supported by the fact that the Smoothened-Hedgehog agonist (SAG) has been shown to play a potential neuroprotective effect mediating the activation of the 11ß-hydroxysteroid dehydrogenase type 2 pathway (11ßHSD2). 11ßHSD2, is a NAD-dependent high-affinity enzyme highly expressed in the placenta and the developing CNS (particularly in the GCPs) mainly involved in the local metabolic inactivation of endogenous glucocorticoids, such as prednisolone and corticosterone (Heine et al., 2011; Nguyen et al., 2018).

#### Pain and Opioids

Preterm infants often experience pain during diagnostic and therapeutic procedures in NICU. Therefore, the use of opioids is quite common in these infants. Recent data have established a correlation between pain, cerebellar underdevelopment, and opioids. Ranger et al. (2015) revealed a significant reduction in the posterior VIIIA and VIIIB lobules of the cerebellum in brain MRI scans of a group of 56 very preterm children at school age who had suffered neonatal procedural pain. Moreover, decreased cerebellar volume has been reported in a cohort of preterm infants treated with morphine (Zwicker et al., 2016). A higher rate of cerebellar injuries and lower cerebellar diameters emerged in another study on preterm infants exposed to fentanyl (McPherson et al., 2015). Furthermore, animal models emphasized the potential detrimental effect of opioids on neurodevelopment. A study carried out by Sabir et al. (2018) showed fentanyl induced apoptosis of the Granule cells of the internal layer in 13 healthy newborn pigs. Aboulhoda and Hassan (2018) detected a relationship between tramadol administration during pregnancy, oxidative stress, and structural abnormalities on the post-natal cerebellar cortex in a group of rats. Finally, another study demonstrated the possible effects of opioids administered during the developmental period on the NMDA receptor (NMDAR) expression and function, exploiting animal models. In particular, it showed how rat cerebella continuously exposed to opioids during the prenatal age may present an opioid-induced reduction of the NMDAR subunit GluN2B (the subunit primarily expressed prenatally in rodents) during the first 3 weeks after birth. This finding supports the idea that NMDAR might be an important target of opioids, especially during neurodevelopment, being potentially involved in their neurotoxic effects and long-term detrimental consequences (Fjelldal et al., 2019).

#### Inadequate Nutrition

Nutrition plays an important role in general growing processes, and CNS structures development, such as the cerebellum. Although infant nutrition is often taken into account among the variables influencing neurodevelopment, especially in preterm infants, a limited number of studies have addressed this issue so far. Limperopoulos et al. (2005) demonstrated a significant correlation between decreased cerebellar volumes in prematures and clinical parameters such as head circumference and weight. This finding indirectly supports the importance of an appropriate nutrition of the baby for cerebellum development, especially in the early postnatal period (when the premature cerebellum is still in the midst of its development). Similar conclusions have been recently described by Coviello et al. (2018), who reported in a cohort of 131 infants, born under the 31st week of gestation and investigated at term equivalent age, a significant correlation between a balanced and normocaloric diet of the baby and larger volumes of CNS structures, including the cerebellum. They also demonstrated a positive correlation between infant nutrition, white matter maturation at term equivalent age, and better neurodevelopmental outcomes at 2 years’ corrected age. Accordingly, Choudhri et al. (2014) demonstrated smaller brains, including cerebellum, and an impaired neurodevelopment in preterm pigs fed via parenteral nutrition. Finally, inadequate maternal nutrition during the gestational age has been linked to an abnormal expression of the enzyme regulating the metabolism of maternal cortisol, namely the placental steroid dehydrogenase, resulting in an excessive cortisol exposure for the fetus (Volpe, 2009). This data appears particularly interesting in the light of the previously mentioned effects of glucocorticoids on the preterm cerebellum. Interestingly, Koning et al. (2017) carried out an observational study highlighting an association between higher maternal BMI and decreased cerebellar growth trajectories, probably due to various mechanisms (i.e., dietary intake, nutritional status, chronic inflammation and oxidative stress). In conclusion, though the exact mechanisms through which nutrition affects brain development remain largely unexplained, it appears that an adequate intake of macronutrients and calories for the mother (during pregnancy), as well as for the infant (since the first moments of postnatal life), play an important role in brain development, certainly worthy of further investigation (Volpe, 2009).

#### Infection/Inflammation and Hypoxia-Ischemia

Prenatal and perinatal infection/inflammation may play a central role in the etiopathogenesis of CH. Evidences of congenital cytomegalovirus infections and poor neurodevelopmental outcomes have been commonly reported, often in the presence of structural CNS lesions, including CH (Smithers-Sheedy et al., 2014; Nishida et al., 2020). Lee et al. (2014) retrospectively studied a cohort of 155 preterm babies, detecting a significant correlation between the presence of necrotizing enterocolitis with sepsis and decreased transcerebellar diameters. Ranger et al. (2015) demonstrated that other factors, including infections, may generate CH in preterm infants beyond neonatal procedural pain. A direct link between animal parvovirus infections and extensive injuries of the cerebellum, including CH, has been documented in numerous species (Aeffner et al., 2006; Marusak et al., 2010; Wünschmann et al., 2020), while little is known when it comes to humans. Grant et al. (2009) showed human parvovirus in the human cerebellum, suggesting a possible role in cerebellar injuries. Moreover, Sanapo et al. (2017) have recently described a case of congenital parvovirus infection associated with bilateral cerebellar hemispheres and inferior vermis hypoplasia, further suggesting a possible involvement of parvovirus in brain pathology. Indeed, the relationship between immune-inflammatory reactions and CNS dysfunctions has been increasingly reported due to the detrimental action of free radicals, cytokines, and several neurotoxic factors. In this regard, systemic inflammation in fetal sheep has been demonstrated to cause disruptive lesions of the cerebellum, throughout different mechanisms, such as an abnormal activation of microglia and apoptosis of Purkinje cells (Hutton et al., 2014). Hypoxia-ischemia plays a potential role in cerebellar underdevelopment mediated by the effects of mechanical ventilation, patent ductus arteriosus, early intubation, and catecholamine treatment, leading to decreased cerebellar volumes in prematures (Volpe, 2009; Gano and Barkovich, 2019). Specifically, GCPs seems to be a potential target of hypoxia-ischemia (Volpe, 2009).

#### Cerebral Brain Injuries (Crossed Cerebrocerebellar Diaschisis)

The experienced use of cranial ultrasound and early brain MRI scans allowed to identify supratentorial injuries causally related to CH in those infants presenting without direct cerebellar lesions (Gano and Barkovich, 2019). Overall, it appears that brain injuries, such as non-cystic periventricular leukomalacia, periventricular hemorrhagic infarction, and posthemorrhagic hydrocephalus, may be responsible for a crossed cerebro-cerebellar diaschisis (Volpe, 2009). In other words, the damage involving supratentorial regions may indirectly induce the impairment of the development of the downstream pathways with consequent dysfunction in neural networks also located at distance, such as the contralateral cerebellum (Patay, 2015). Conversely, it is of interest that a primary lesion of the cerebellum has been demonstrated to impact the brain’s development or functioning. This is the case, for example, in cerebellar mutism, occurring after postoperative posterior fossa syndrome. This particular condition appears to be a reverse form of diaschisis, namely cerebello-cerebral diaschisis, in which damage, primarily involving the cerebellum and its efferent networks, eventually results in a global supratentorial cortical dysfunction (Mariën et al., 2009). Specifically, cerebellar mutism seems to be strictly related to a trans-synaptic cerebral cortical dysfunction presenting with supratentorial hypoperfusion, mostly in the frontal regions, due to postsurgical damage to efferent cerebellar pathways (Patay, 2015). Although this syndrome’s pathophysiology and anatomic basis are not completely known, an impairment of the dentato-thalamo-cortical pathway is thought to play a central role (Morris et al., 2009). Moreover, vasospasm of vessels supplying the deep cerebellar nuclei and the cerebello-thalamic fibers, due to different perioperative factors, has been suggested as a potential cause of reversible hypoperfusion and ischemia (Catsman-Berrevoets, 2017). Furthermore, numerous neuroimaging findings support the correlation between abnormal supratentorial perfusion patterns and cerebellar mutism syndrome, strongly supporting the current accepted notion of cerebello-cerebro diaschisis as the underlying pathomechanism for this peculiar clinical entity (Miller et al., 2010; Patay, 2015). The demonstration represents well another worthwhile example of cerebello-cerebral diaschisis, that large hemorrhages of the cerebellum not only result in a focal atrophy, but they may also correlate to a decreased volume of contralateral cerebral regions (Limperopoulos et al., 2014). On the whole, though much more has yet to be unraveled on the topic, remote trophic transneuronal signaling likely underlies these mechanisms (Volpe, 2009).

**FIGURE 1:**
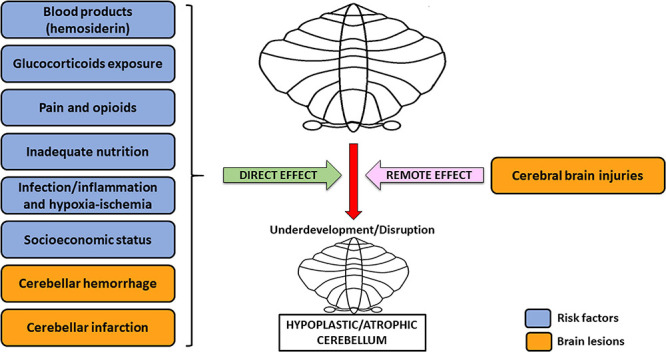
Risk factors and brain lesions leading to cerebellar underdevelopment/disruption. The risk factors nowadays acknowledged for cerebellar underdevelopment/disruption are: blood products (hemosiderin), glucocorticoids exposure, pain and opioids, inadequate nutrition, infection/inflammation, and hypoxia-ischemia. All of these, together with cerebellar hemorrhage and infarction (possible injuries affecting the cerebellum), usually act through a direct effect, potentially leading to cerebellar hypoplasia/atrophy. However, this latter condition may also be the result of cerebral brain injuries, which cause dysfunction in a distant, though neurally connected area of the brain, i.e., the contralateral cerebellum (remote effect) (Volpe, 2009; Pierson and Al Sufiani, 2016).

Overall, the etiology of cerebellar underdevelopment can be traced back to two groups of mechanisms, that are (1) direct effects and (2) remote effects, which may act alone or concomitantly (Volpe, 2009), potentially resulting in greater damage on the developing cerebellum (Figure 1). This is the case of CH generated by a crossed cerebro-cerebellar diaschisis from loss of contralateral connections, in which further damage is produced by a concomitant ventricular hemorrhage (Tam, 2018). As a rule, blood products accumulation, glucocorticoids, opioids, inadequate nutrition, infections/inflammations, and hypoxia-ischemia may directly cause a detrimental effect on the growing cerebellum. Conversely, cerebral injuries (crossed cerebro-cerebellum diaschisis) act *via* an indirect remote effect, disrupting trans-synaptic interconnections, even at distance (Volpe, 2009; Pierson and Al Sufiani, 2016).

#### Socioeconomic Status

Besides the aforementioned risk factors, it is noteworthy mentioning that cerebellar normal development has been related to socioeconomic status (Tam, 2018). The literature suggests an association between poor socioeconomic conditions and higher inflammatory and cardiometabolic risks, and poor neurocognitive outcomes. Interestingly, Cavanagh et al. (2013) carried out a study on a group of healthy males with a deprived socioeconomic status, detecting lower cerebellar gray-matter volumes. Even though these findings do not directly refer to prematurity, socioeconomic status could represent an additional risk factor likely offering new investigation fields.

### Destructive Cerebellar Lesions

Focal or diffuse brain injuries in preterm infants have long been widely described as potential causative factors of subsequent neurodevelopmental impairment. Awfully, despite the reduction of the occurrence of the worst patterns of lesions, a correspondent decrease in preterm neurodevelopmental disabilities has not been shown (Gano and Barkovich, 2019). Given these assumptions and the raised interest in the cerebellum involvement in motor and non-motor functions, such as cognitive, affective, and behavioral ones, a correlation between destructive cerebellar lesions and their consequences has been extensively studied. This is particularly true in the light of the improved care in NICU of the at-risk newborns leading to the increased survival rate of preterm infants (Tam, 2018).

Cerebellar focal injuries are commonly considered as premature birth complications and they generally consist of hemorrhages and infarctions, though a clear distinction between them is hard to be made since they frequently coexist in the same patient. These lesions are usually observed in the inferior lobes of the cerebellum, an area supplied by the posterior inferior cerebellar artery, leading to the speculation that an immature development of this vessel might be the origin of both damages (Pierson and Al Sufiani, 2016).

#### Cerebellar Hemorrhage

Cerebellar hemorrhage is the most explored injury affecting the cerebellum in preterm newborns with incidence ranging from 2.2 to 37% (Boswinkel et al., 2019; Gano and Barkovich, 2019). The wide range of incidence across the studies may rely on the different employed diagnostic tools: cranial ultrasound is feasible and reliable in routine preterm care in NICU, and frequently used to evaluate the posterior fossa serially, but it mainly detects wider lesions. In contrast, brain MRI is sensible to catch spot injuries smaller than 4 mm, though its application is often limited to cases presenting with evident neurological signs, with heterogeneous protocols from center to center (Boswinkel et al., 2019). In regards to cranial ultrasound, different acoustic windows can be explored, from the anterior fontanel to the posterior one, although the mastoid fontanel is commonly considered the best option to visualize cerebellar and brainstem lesions (Snyder et al., 2018). Recently, a suboccipital access through the foramen occipital magnum has been considered for its higher resolution of the cerebellar parenchyma, allowing to detect smaller lesions and the possibility to compare both hemispheres at the same time (Muehlbacher et al., 2020).

Cerebellar hemorrhage is a well-known complication of premature birth with a higher risk of incidence in children born before 28 weeks of gestational age and a birthweight inferior to 750 g. This is even truer in premature newborns with worse adaptation to extrauterine life (Volpe, 2009; Dijkshoorn et al., 2020). Other noteworthy risk factors include preeclampsia, traumatic delivery, sepsis, prolonged mechanical ventilation, patent ductus arteriosus, and hemodynamically significant hypotension (Boswinkel et al., 2019; Gano and Barkovich, 2019; Garfinkle et al., 2020).

Although CBH is commonly associated with multifactorial etiology, cerebrovascular alterations have been mostly emphasized, suggesting the impaired autoregulation of the cerebral circulation and consequent treatment being the principal etiological factors (Tam, 2018; Boswinkel et al., 2019). This autoregulatory function develops progressively between the 23rd and the 33rd week of gestation (Rhee et al., 2016). Therefore, preterm infants may present with anatomically incomplete and underdeveloped cerebral vasculature, not yet fully able to autoregulate, determining an increased risk of brain injuries due to cerebral blood flow fluctuations (Rhee et al., 2018). From the microanatomic perspective, the cerebellar zone mostly involved in CBH is the external granule cells layer, since it is highly vascularized and contains overall the highest number of cells among the nervous system. CBH may be consequent to a vulnerability of the newly formed vessels in the external granule cells layer supplied by the posterior inferior cerebellar artery. Specifically, it appears that the fast angiogenesis taking place during the third trimester in the germinal matrix of the cerebellum (located in the roof of the fourth ventricle and the external granule layer) may generate vessels that are still immature. These vascular structures cannot sustain these cerebral flow fluctuations; hence they may be more prone to insults (Volpe, 2009). Accordingly, Pierson and Al Sufiani (2016) stated that the recurrent association between CBH and intraventricular hemorrhage (IVH) relies on a shared pathogenetic mechanism, by which fluctuations of the blood pressure may increase hemorrhagic risk in both districts. The authors also proposed that CBH might result from a chronic process, with multiple hemorrhagic events occurring over time with single lesions merging into larger ones. Moreover, CBH location appears to be related to the origin of the bleeding, being the germinal matrix of the ventricular zone near the fourth ventricle mainly implicated in vermian hemorrhages, and the one located in the external granule layer more likely associated with focal unilateral CBH (Volpe, 2009).

**FIGURE 2:**
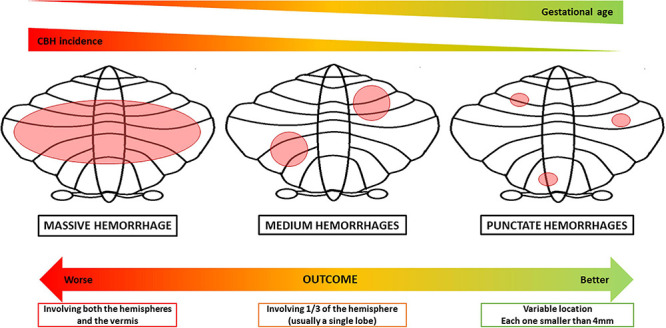
Types of cerebellar hemorrhage in relation to possible timing and outcomes. The picture shows the possible correlation between lower gestational ages of birth and higher incidence of larger hemorrhages, concerning outcomes. Massive hemorrhage usually involves both the hemispheres and the vermis and can be related to supratentorial lesions, i.e., IVH. It has the worst outcome, leading to generalized cerebellar atrophy and to significant neurological sequelae, presenting the highest mortality rate. Medium hemorrhages involve only one-third of the cerebellar hemisphere, hence usually a single lobe (i.e., anterior or posterior lobe). It frequently evolves in focal atrophy and a variable neuropsychiatric outcome, typically milder than the massive one. Punctate hemorrhage is smaller than 4 mm. Generally, it does not lead to cerebellar hypoplasia and presents minimal neurological abnormalities, depending on the lesion site (Volpe, 2009; Zayek et al., 2012; Boswinkel et al., 2019; Gano and Barkovich, 2019).

Cerebellar bleeding may involve different parts of the cerebellum and, according to its extension, it has been classified as massive, medium, and punctate CBH (Boswinkel et al., 2019). Since it has a high mortality rate, larger CBH may be detected as an autoptic finding. It usually involves both cerebellar hemispheres and can be related to supratentorial lesions, as the IVH, leading to obstructive hydrocephalus (Tam, 2018). The outcome of the massive bleeding typically includes generalized atrophy with severe neurological sequelae, i.e., cerebral palsy. Vermis involvement is frequently associated with social and behavioral disorders, such as those typically detectable in the autism spectrum disorder (Limperopoulos et al., 2007). On the contrary, punctate hemorrhages, namely hemorrhages smaller than 4 mm, do not present with acute clinical signs and usually do not develop in cerebellar atrophy. It is commonly accepted that these lesions result in better neurodevelopmental outcomes, with minimal neurological abnormalities in tone, strength and reflexes, and cognitive level varying from normal to lower based on the cerebellar region injured (Boswinkel et al., 2019; Gano and Barkovich, 2019). CBHs involving only one-third of the cerebellar hemisphere are named medium or limited hemorrhages: they frequently involve only a lobe, developing into focal atrophy and leading to a milder neuropsychiatric outcome than the one caused by the massive CBH (Volpe, 2009; Boswinkel et al., 2019). Given the above and given the correlation between lower gestational age of birth and higher incidence of CBH and IVH reported by Zayek et al. (2012), it is presumable that preterms born at lower gestational ages may present with larger hemorrhages with worse outcomes (Figure 2).

#### Cerebellar Infarction

The cerebellar infarction is another type of direct lesion of the cerebellum in preterm infants and, as the CBH, it is considered a complication of extreme prematurity. Both the CBH and the infarction seem to be related to the same pathogenetic mechanism, underlying the vessel walls’ immaturity due to the rapid angiogenesis (Volpe, 2009). As mentioned above, the newly formed vessels seem to be affected by fluctuations in cerebral blood flow, causing several brain injuries. This is even more interesting in the light of the fact that blood pressure is lower in preterm infants compared with term ones and, especially in prematures with hypotension, cardiac cycle changes can influence cerebral blood flow, often being absent during diastole (Rhee et al., 2018).

Additionally, neonatal respiratory distress syndrome, a common condition in premature babies, and its treatment through non-invasive respiratory support, may affect cerebral perfusion. A recent study on nasal continuous positive airway pressure (nCPAP), one of the most frequent ventilation methods used in NICU, analyzed the effects of high pressure on cerebral hemodynamics, stating that cerebral perfusion remains relatively stable when the pressure is set between 4 and 8 cmH_2_O (Zhou et al., 2020).

As for CBH, the most affected area is the inferior cerebellum, supplied by the posterior inferior cerebellar artery, and an ischemic infarction possibly evolves into a hemorrhagic lesion (Mühlbacher et al., 2018). The hypoxic-ischemic insult determines a loss of neurons in the internal granule layer, resulting in a focal atrophy of the cerebellar parenchyma (Pierson and Al Sufiani, 2016). Even more than CBH, the cerebellar infarction is related to supratentorial white matter abnormalities, suggesting a common pathogenesis (Volpe, 2009; Suppiej et al., 2015).

## Neurodevelopmental Outcomes

According to the cerebellum’s central role in sensorimotor control, posture and tone, executive functions, language, and behavioral regulation, it should not surprise that a significant correlation between cerebellar lesions and impairment of the neurodevelopmental outcome, especially in preterm infants (Brossard-Racine et al., 2015). As previously discussed, impaired cerebellar development, with its adverse consequences, may originate both from direct and indirect injuries, with or without a structural correlate.

Regarding direct injuries, many studies have focused on the neurodevelopmental outcomes deriving from hemorrhages. It appears that the larger is the hemorrhage, the worst are the effects on motricity, cognitivity, language, and behavior (Gano and Barkovich, 2019). Motor impairments are mainly encompassed by cerebral palsy, the severity of which appears to be related to procedures of shunt positioning, and, in a direct proportionate manner, to the grade of IVH, to the extent of white matter damage, and to the size of ventricles (Valdez Sandoval et al., 2019). A study conducted by Tam et al. (2011) reported alterations in muscle tone, strength, and reflexes in association with small CBHs, detectable only via MRI rather than cranial ultrasound. A various degree of motor delay has been previously reported in the literature (Gano and Barkovich, 2019) with more severity in preterm infants who present with cerebellar hemorrhagic injury concomitant to supratentorial parenchymal lesions (Limperopoulos et al., 2007). Rarely, CBH may also result in limited and specific motor dysfunctions. In this regard, a late preterm infant with a hemorrhagic lesion in the right cerebellar hemisphere and the vermis, with midline shift and intraventricular bleeding, presented with right peripheral facial palsy at 24 h of life (Coviello et al., 2020). About the non-motor domains, Limperopoulos et al. (2007) reported severe impairments in infants with isolated cerebellar hemorrhagic injuries, particularly involving communicative (in terms of both expressive and receptive language) and social-behavioral skills, including internalizing and externalizing problems as well as autistic features. A large study on 397 extremely preterm born children revealed pathological outcomes in social-emotional competences (26%) significantly related to the presence of cerebellar lesions on near-term MRI (Duncan et al., 2019).

Depending on the affected cerebellar region, infarction has also been related to various degree of neurodevelopmental sequelae, i.e., cerebral palsy, microcephaly, cognitive and language delay, ataxia and other sensorimotor abnormalities (Pierson and Al Sufiani, 2016; Mühlbacher et al., 2018). Patients with cerebellar underdevelopment, either with or without structural damages, have been shown to have several neurodevelopmental impairments. A significant correlation between CH with poor motor performances and cognitive defects has been reported, showing a lifespan course up to adulthood (Gano and Barkovich, 2019; Tam et al., 2019). Mixed spastic–ataxic–dyskinetic type of cerebral palsy with severe cognitive deficits have been reported in a case-control study carried out on prematures presenting with a disrupted cerebellar development (Messerschmidt et al., 2008). Ranger et al. (2015) demonstrated that a reduction of the posterior VIIIA and VIIIB lobules of preterm children exposed to opioids is significantly related to poorer outcomes in terms of cognition and motor/visual integration. Poor motor and cognitive outcomes at 18 months have been shown in the cohort of preterm patients exposed to morphine studied by Zwicker et al. (2016). Lind et al. (2011) showed in a cohort of 164 preterm patients a correlation between reduced total brain volume, including cerebellum, and larger ventricles, with poor neurological performance at 2 years of corrected age. Another study on a vast cohort of very preterm infants, evaluated at term equivalent age and at 7 years, demonstrated a correlation between CH and lower Intelligence Quotient (IQ), math computation and motor abilities (Anderson et al., 2017). Lower IQ was also related to persistent CH in preterm adolescents (Allin et al., 2001). In contrast, other studies failed to find significant correlations between motor and/or cognitive performances and cerebellar volume in prematures investigated at 2 and 5 years of age (Shah et al., 2006; Lind et al., 2010). Therefore, although during the last decade the role of cerebellar injuries in the neurodevelopmental outcome of preterm infants has been widely investigated, much more has yet to be unraveled on the topic, and further studies are needed.

## A Brief Overview on Early Intervention

The recovery after cerebellar damage is usually slow, partial, and mostly depends on the lesions’ site and size (Kelly and Shanley, 2016). Despite several reports showing abnormal neurodevelopmental outcomes in preterm infants with both cerebellar dysmaturative and destructive lesions, less evidence is available for their early detection at long-term follow-up. Standardized neurological scales (i.e., Hammersmith Infant Neurological Examination) are extremely useful to assess neurological deficits during the first year of life. However, previous reports failed to show specific score range in patients with cerebellar malformations or injury who will later develop ataxia (Novak et al., 2017).

To date, no standardized protocols have been established to treat infants with cerebellar lesions specifically; therefore, more evidences on early neuromotor patterns are needed in order to provide targeted interventions. Kelly and Shanley (2016) proposed various rehabilitation strategies (such as strength and balance training, use of environmental cues, walking aids, compensatory head fixing, and retraining of specific abilities) targeting symptoms deriving from site-specific lesions of the cerebellum. In patients with cerebellar mutism syndrome, cognitive potentiation, physiotherapy, occupational, and language therapy are useful tools for an early recovery of motor and communicative functions (Catsman-Berrevoets, 2017). In the light of the frequent concomitant involvement of the supratentorial regions and given that a specific protocol of rehabilitation in cerebellar injuries has not been reported, appropriate treatment may also be chosen accordingly to previous evidences (Novak et al., 2017). Encouraging data are reported for constraint-induced movement therapy (CIMT), both prolonged and intermittent, as a feasible and effective treatment for hemiplegic cerebral palsy (Christmas et al., 2018). Conversely, CIMT has proved less efficacious than the same amount of upper limb therapy with no restraint (Chiu and Ada, 2016). Other types of interventions, such as gait and velocity trainings, electromyographic biofeedback training, and whole-body vibration, have been attempted to target the gait speed (Moreau et al., 2016). Slackline training has been reported as a useful tool to implement static postural control and motor skills (González et al., 2020). Moreover, the virtual reality training offers promising results in coordination and sensory-motor functions, and participation in daily living activities in multiple settings (Lopes et al., 2020). Patients with cerebellar damage may present impairments of some cranial nerve functions (i.e., swallowing and oculomotor coordination), based on which appropriate treatment can be addressed. Fucile et al. (2012) found that premature infants may benefit from oral, tactile/kinesthetic, and combined (oral plus tactile/kinesthetic) interventions, with better sucking skills and swallow-respiration coordination. Also, a multisensory early intervention program targeting visual functions may positively impact the neuro-visual outcome of preterm infants (Fontana et al., 2020).

## Discussion

During the last decades, more and more attention has been paid on the cerebellum. From the initial assumption of a mere role in motor coordination, new evidences have been continuously emerging on cerebellum involvement in a multiplicity of other functions (i.e., cognition, behavior, language, memory, and learning) (Salman and Tsai, 2016).

The human cerebellum develops over a long period of time; however, most of its volume increases during the last trimester of pregnancy. Therefore, this is considered a critical period for cerebellar maturation, even more when prematurity with all its contingencies occurs (Volpe, 2009; van Essen et al., 2020).

Here, we discussed the cerebellar neuropathology of preterm infants. Particularly, we retraced cerebellar development and analyzed the main factors entailed in the genesis of cerebellar underdevelopment and cerebellar destructive lesions in preterm infants, finally giving a brief overview of the potential neurodevelopmental outcomes and early intervention.

Even though the implications of encephalopathy of preterm have already been demonstrated, several undefined questions need to be answered.

First of all, the specific early clinical correlates of cerebellar prenatal and perinatal damages are yet to be well-defined by the most validated neurological examination scales in infancy. Second of all, few data on an early intervention for the preterm infants presenting with cerebellar injuries have been reported.

In light of this, we reckon that further studies specifically addressing these unsolved issues are needed. For instance, it would be of great interest and help for the clinical practice to analyze the correlation between clinical signs of suspect of cerebellar impairment, investigated through standardized neurological scales, and neuroimaging cerebellar abnormalities. This may help detect some red flags specifically related to cerebellar lesions, establish standardized tools of evaluation, and avoid the performing of useless and expensive examinations. More evidence on early neuromotor patterns would also be fundamental in providing targeted early intervention protocols and better assessing the prognosis. Finally, given the currently accepted notion that the cerebellum plays an important influence on non-motor functions (such as behavioral and social abilities), the follow up-programs of preterms with early cerebellar lesions should rightly include a systematical assessment of these skills.

Furthermore, other unsolved questions need to be answered regarding some of the above-mentioned causal factors of CH. For instance, as previously discussed, the molecular basis of glucocorticoids-induced brain injury has not been completely unraveled (Heine and Rowitch, 2009). A potential mechanism through which glucocorticoids may interfere with normal neurodevelopment appears to be via the inhibition of the Sonic-Hedhehog-Smoothened signaling, involved in the proliferation of the GCPs (Heine and Rowitch, 2009). Moreover, knowing the exact degree of causality and mechanisms through which molecules as glucocorticoids may actually act and cause CNS lesions in preterm would provide new insights on the topic and modify and improve the prenatal care protocols. The same goes for other modifiable factors previously mentioned, such as nutrition (both infant and maternal ones) or infections. More thorough and systematic research on the topic may unravel the complex relationship between cerebellum and prematurity and help identify clear causality links, eventually allowing the modification of clinical and therapeutic protocols. Finally, comparative case-control studies may be of interest in detecting some protective factors too.

In conclusion, the complex relationship between cerebellar neuropathology and prematurity still gives rise to numerous and unsolved questions. Further studies on early detection, both clinically and neuroradiologically based, and on individually tailored intervention strategies or on the causality-link of numerous factors involved in cerebellar lesions need to be addressed in wide and gestational age-matched populations.

## Author Contributions

GD and AN designed and directed the project. GS and GA drafted the manuscript and designed the figures. LV, GQ, AC, and EG helped to supervise the project. All authors discussed the results and contributed to the final manuscript.

## Conflict of Interest

The authors declare that the research was conducted in the absence of any commercial or financial relationships that could be construed as a potential conflict of interest.
